# Supplementary material for: Risk factors for early PICC removal: A retrospective study of adult inpatients at an academic medical center
Source: PLoS One. 2022 Jul 8;17(7):e0264245. doi: 10.1371/journal.pone.0264245 (PMC9269940; doi:10.1371/journal.pone.0264245)
Supplement: Attachment — Submitted filename: PLOS ONE Response to Reviewers 2 (002).docx [file pone.0264245.s004.docx]

June 23, 2022

Marc O. Siegel, MD

Academic Editor

PLOS ONE

2150 Pennsylvania Ave NW

Washington, DC, 20037

Dear Dr. Siegel,

Thank you for reviewing and considering our manuscript. We appreciate the questions and suggested changes brought forth by the reviewers. We are submitting a tracked changes version of our manuscript and a clean copy.

Please see below for the reviewer comments and our response.

Scientific comments:
1. Abstract Results: “Patients with PICCs whose primary service was a medical subspecialty were independently at higher risk of early removal” This result should be updated according to new results.

- **We have updated the abstract results.**

2. Results: “In the 1/1/2017 to 12/31/2017 data set, female sex was associated with earlier PICC removal.” In this sentence, there were no p-values. Meanwhile, in table 3, female sex is not significantly associated with early removal (p=0.14). P-value of dataset of 2017 was not given. Please, clarify what lead to these results.

- **We have removed this line since this was a remnant of the previous analysis.**

3. Results: “Approximately one-third of PICCs were placed in patients who were in an intensive care unit” In table 1, 30.8% of patients were medicine subspeciality. ICU was 11%. There seems to be a confusion.

- **We have changed it to the correct number (11%) for ICU.**

4. Results: “From 1/1/2017 through 12/31/2017, 141 PICCs were removed within five days of insertion. Among these 141 PICCs, 105 (74%) were not removed due to complications.” This sentence is inappropriate. “105 (74%) were removed from unrelated to complications” would be right.

- **We have made this correction.**

5. Results: Table 1 shows total patient number as 7358. It should be clarified which analysis was applied for 7358 patients’ group. In results section, there seems to be no analysis on 7358 Pts. If there is no analysis on 7358, table 1 should include data of 5348 patients.

- **We agree that the table and data is confusing with the 7358 total PICCs and the analysis done on the 5348 PICCs removed. We have simplified Table 1 to include just the 5348 patients with PICCs removed.**

Grammar/style comments

6. Abstract Results: Between 1/1/17 and 5/4/2020 -> Between 1/1/2017 and 5/4/2020

- **We have made this correction.**

7. Discussion: Aside from BMI in the univariable analysis, comorbidities as a group were not associated with earlier PICC removal. BMI has previously been shown to be associated with PIC complications, such as CLABSI.[15] -> PICC

- **We have made this correction.**

8. Table 3. Female 0.87 (0.7201.05) -> 0.87 (0.72-1.05)

- **We have made this correction.**

Leonard A. Mermel, DO, ScM, AM (Hon), FSHEA, FIDSA, FACP
Professor of Medicine, Warren Alpert Medical School of Brown University
Medical Director, Department of Epidemiology & Infection Prevention, Lifespan Hospital System Adjunct Clinical Professor, University of Rhode Island College of Pharmacy
